# A systematic review of the spectrum of magnetic resonance imaging features of degenerative thoracic myelopathy

**DOI:** 10.1007/s10143-026-04535-z

**Published:** 2026-10-26

**Authors:** Eleanor White, Amir Rafati Fard, Jay Wardropper, Renuka Chintapalli, Edwin Koubeh, Hariesha Pathmaraj, Sruthi Ranganathan, Tanzil Rujeedawa, Chandan Singh Sekhon, Jibin J. Francis, Benjamin M. Davies, Mark R. Kotter, Oliver D. Mowforth

**Affiliations:** 1 School of Clinical Medicine, University of Cambridge, Cambridge, UK; 2 Division of Neurosurgery, Department of Clinical Neurosciences, University of Cambridge, Cambridge, UK

**Keywords:** Thoracic myelopathy, Disc herniation, Spondylosis, Spinal stenosis, Spinal cord injury, Magnetic resonance imaging

## Abstract

Purpose. Degenerative thoracic myelopathy (DTM) encompasses a range of degenerative pathologies associated with symptomatic spinal cord compression. Diagnosis is achieved through clinical history, examination, and magnetic resonance imaging (MRI). A comprehensive review of the MRI features of DTM and their clinical significance is currently lacking. The objective was therefore to synthesise current evidence. Methods. A systematic review was performed adhering to PRISMA guidelines. MEDLINE and Embase were searched. Screening, data extraction and risk of bias assessments were performed in duplicate. A qualitative synthesis was conducted following the Synthesis Without Meta-Analysis (SWiM) guidelines. Results. Of 7937 records, 59 papers studying 2940 patients satisfied inclusion criteria, including one cross-sectional and 58 cohort studies. DTM was most common in the lower thoracic spine (62.5%, 880/1409), with a roughly equal prevalence in the upper (18.2%, 256/1409) and middle regions (19.4%, 273/1409). Ossification of the ligamentum flavum (OLF) was the most common pathology (71.4%, 2065/2893), followed by intervertebral disc herniation (20.1%, 582/2893) and ossification of the posterior longitudinal ligament (OPLL) (19.7%, 569/2893). Measurements of spinal canal compromise were described in 55.5% of patients (1631/2940) and spinal cord signal change in 30.0% (881/2940). Spinal canal compromise, spinal cord signal change, OLF, OPLL and multilevel disease were associated with greater DTM severity and poorer functional recovery. Conclusion. Ligamentous pathologies are significantly more prevalent than disc related pathologies in the DTM literature. Spinal cord signal change and extent of spinal canal compromise correlate with clinical outcomes. Standardisation of terminology for imaging features and study design are required to improve future research.

**Supplementary Information:**

The online version contains supplementary material available at https://doi.org/10.1007/s10143-026-04535-z.

## Introduction

Degenerative thoracic myelopathy (DTM) is an umbrella term for degenerative thoracic spinal pathology associated with symptomatic spinal cord compression [67]. Aetiological pathologies include spondylosis, degenerative disc disease and spinal ligament hypertrophy, calcification and ossification.

Similarities in the presentation and management of these pathologies support the use of the term DTM as an overarching terminology to promote consistency in clinical care and research, as mirrored by the term degenerative cervical myelopathy (DCM). The better-researched entity of DCM has been proposed to arise due to abnormal mechanical loading of the cervical spine over time, with both environmental and genetic contributions [17, 63]. However, given the differing mechanical properties of the thoracic spine, which likely result in distinct mechanical stress patterns, the nature of degenerative pathology causing compression in DTM may differ, with potential implications for diagnosis and management.

Symptoms and signs of DTM include leg and back pain and neurological deficits including motor, sensory, bowel and bladder dysfunction [5]. The impact on patients is substantial, with studies demonstrating that DTM patients experience an overall poorer satisfaction rate after surgery than those undergoing surgery for cervical or lumbar disease [30]. The disability experienced by those with DTM and the resultant reduction in productivity implies that this diagnosis incurs a substantial economic burden, as seen in DCM [18].

The progressive nature of DTM necessitates prompt diagnosis and management [45, 67]. Delays risk irreversible neurological damage [1, 74]. However, several challenges impede timely diagnosis. Firstly, an overlap of symptoms with disease at other levels of the spine means that thoracic myelopathy is not uncommonly initially mistaken for more common cervical or lumbar pathology [5]. Further diagnostic complexity can also arise from concurrent neurological pathology, such as peripheral neuropathy [23, 51]. Clinical history and examination therefore remain essential in formulating a suspected diagnosis on the basis of neurological symptoms and signs. Thereafter, definitive diagnosis depends on concordant magnetic resonance imaging (MRI) features characteristic of DTM.

The existing literature lacks a comprehensive synthesis of the nature and relative frequency of pathology in patients with DTM and the implications of individual MRI features for prognosis and outcomes. The objective of this review was therefore to address these knowledge gaps by characterising the spectrum and prevalence of causative pathology identified on MRI in patients with DTM and identify features that are important for prognostication and management.

## Methods

### Study design

A systematic review was performed in accordance with the Preferred Reporting Items for Systematic Reviews and Meta-Analyses (PRISMA) guideline [48]. The protocol was registered on PROSPERO (CRD42024518673). Clinical trial number: not applicable.

### Eligibility criteria

The eligibility criteria used to identify relevant studies are outlined in Table 1.

**Table 1:** Inclusion and exclusion criteria for title and abstract screening

Inclusion criteria	Exclusion criteria
Primary study	Review or meta-analysis
Patients with degenerative thoracic myelopathy	Case report, case series (<5 patients) editorial, opinion article, letter or correction
Data on thoracic spinal MRI	Full text unavailable
Human study	
Adult population (>18 years old)	
English language	

### Information sources 

MEDLINE and Embase were search from inception to February 2025 using Ovid (Ovid Technologies, New York, NY, United States). References lists of included studies and relevant review articles were hand searched for additional studies. 

### Search strategy

The search strategy was developed and iteratively refined, including review by a medical librarian at the University of Cambridge (Supplementary Materials, Table 1). To maximise sensitivity, no automated search limits were applied. 

### Selection process

Screening was completed using a two-phase process. Firstly, all primary studies reporting on patients with degenerative thoracic myelopathy were identified to compile a database of all published studies on DTM. Papers were excluded if the title and abstract indicated failure to meet inclusion criteria or satisfied at least one of the exclusion criteria. Seven reviewers (EW, CS, EK, JW, SR, HP, TR) independently performed blinded title and abstract screening using Rayyan (Rayyan Systems Inc., Cambridge, USA). A second phase of screening was conducted within the database of DTM studies by two blinded reviewers (EW and JW) to identify studies that reported on MRI findings. In both phases, a blinded pilot screen of 100 publications was first conducted to ensure concordance between reviewers. Any disagreements after unblinding were resolved by discussion between the reviewers until agreement was achieved. 

### Data collection

Manual data extraction was completed in duplicate (EW and JW) in Microsoft Excel (Version 16.63, Microsoft 365) using a piloted extraction table. 

### Data items

After the second round of title and abstract screening, articles were retrieved for full text screening and data extraction. Data extracted from studies included: author, publication year, study aims, study design, inclusion and exclusion criteria, sample characteristics (size, mean age, age range, biological sex, ethnicity and symptom duration), MRI sequences performed, MRI outcomes assessed, type of surgery and follow-up duration, clinical measures before and after surgery, MRI changes before and after surgery, key associations between clinical measures and MRI changes, relevant statistical analysis, main conclusions and limitations. 

### Risk of bias assessment

The risk of methodological bias in individual studies was examined in duplicate (EW and JW) using the Joanna Briggs Institute (JBI) critical appraisal tools for analytical cross-sectional studies and cohort studies (Supplementary Materials, Figs. 1 and 2) [99]. Criteria 2 and 3 were deemed not applicable for the cohort studies in our review. Each study was assigned a score from 0 (no criteria satisfied) to 8 or 9 (all criteria satisfied for cross-sectional and cohort study checklists respectively), where a ‘Yes’ scored 1, ‘Unclear’ scored 0.5 and ‘No’ scored 0. We trichotomized this ordinal scale to classify the risk of bias as high (scores 0-2.5), moderate (3-5 or 3-6 for cross-sectional and cohort studies respectively), and low (5.5-8 or 6.5-9 for cross-sectional and cohort studies respectively) [65]. 

### Synthesis methods

Due to significant heterogeneity in study design, inclusion criteria, interventions and outcome assessments of included studies, meta-analysis was not possible. A qualitative synthesis without meta-analysis (SWiM) was therefore conducted [13].

## Results

### Study selection

**Fig. 1:**
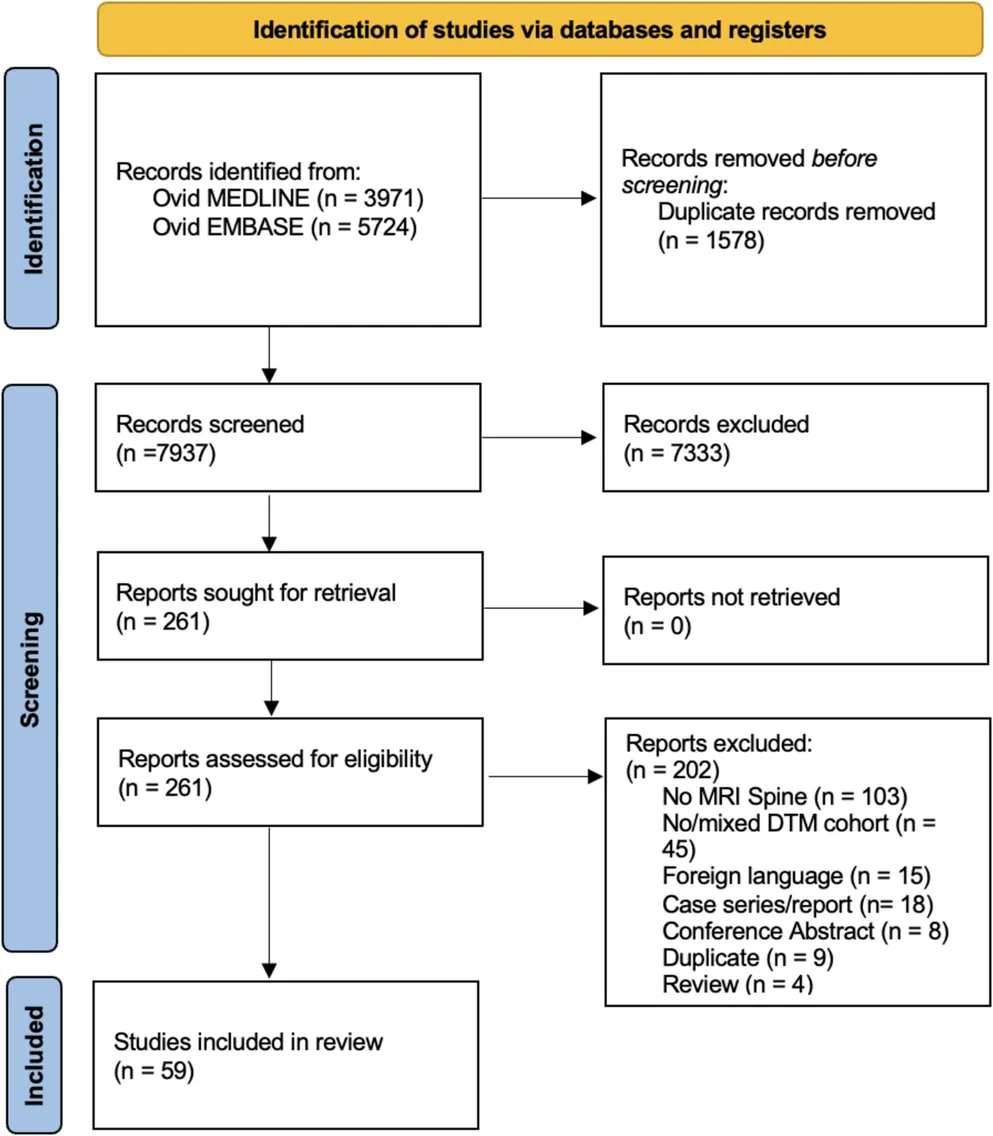
PRISMA flow diagram of study selection

From 7937 results, 59 studies were included in the final analysis. Of these, 58 were cohort studies and one was a cross-sectional study (Fig. 1).

### Study characteristics

The number of patients per study ranged from 7 to 257 with a median of 33 (IQR 20–56). The age of patients ranged from 43.7 to 70 years, with a mean of 56.4 years (SD 5.5 years) and a median of 56.4 years (IQR 52.3–59.7). A total of 57.3% (1686/2940) of patients were male. At least one quantitative measure of symptom severity was reported in 95% (56/59) of included studies. The three remaining studies provided a qualitative description of symptoms [2, 72, 88]. Most included studies (81.4%, 48/59) reported baseline spinal MRI only, with a minority presenting both pre- and post-operative MRI (18.6%, 11/59).

**Fig. 2:**
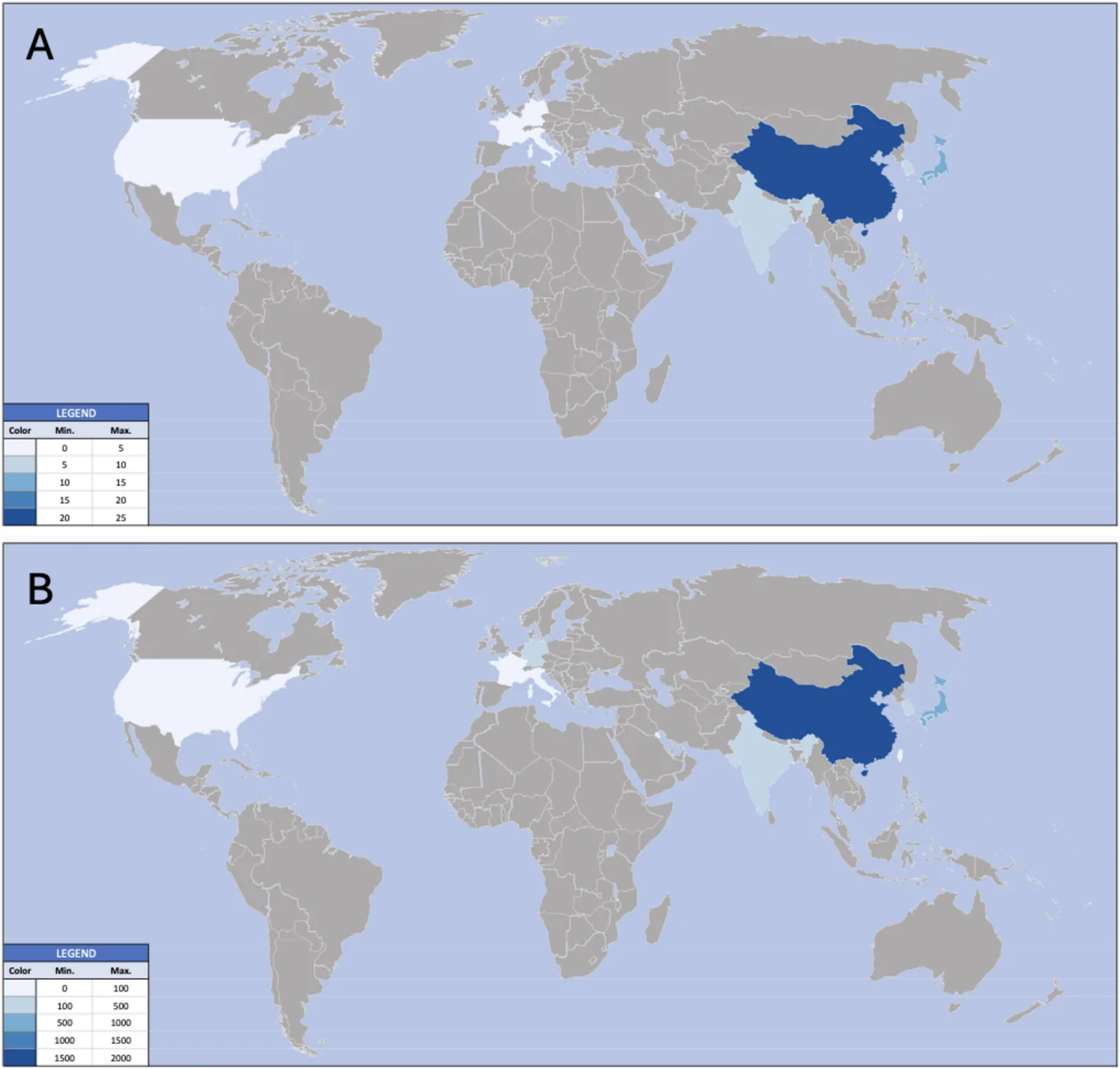
World heatmaps showing research output on MRI findings in DTM by country in terms of (**A**) number of studies and (**B**) number of patients. The higher the output the darker the shade of blue in which the country is depicted. Researchers from China and Japan were the greatest producers of this research, together contributing two thirds of included studies

The majority of studies were performed in the Asian continent (Fig. 2), with nearly half of all included studies emanating from China (42.4%, 25/59), and nearly one quarter from Japan (23.7%, 14/59). Only 6.7% (4/59) of included studies were performed in Europe.

## Spectrum of DTM pathologies

A degenerative pathological finding on MRI to which DTM was attributable was specified for 98.4% (2893/2940) patients. In 10.4% (302/2893) of patients concurrent degenerative pathologies were identified elsewhere in the thoracic spine.

Ossification of the ligamentum flavum (OLF) was the most common finding and was reported in 71.4% (2065/2893) of patients. This was followed by herniated intervertebral discs (20.1%, 582/2893) and ossification of the posterior longitudinal ligament (OPLL) (19.7%, 569/2893). Less common findings included calcified herniated discs (2.7%, 77/2893), posterior osteophytic spurs (0.6%, 17/2893), calcification of the posterior longitudinal ligament (CPLL, 0.3%, 8/2893), spondylolisthesis (0.1%, 4/2893) and calcification of the ligamentum flavum (CLF, 0.03%, 1/2893).

Concurrent degenerative disease in the thoracic spine was present in 49% (281/569) of patients with OPLL, and in 15.4% (319/2065) of those with OLF. Concurrent degenerative disc disease affecting the thoracic spine was seen in 2.3% (13/569) of patients with OPLL, and 3.7% (77/2065) of patients with OLF.

### Anatomical distribution

**Fig. 3:**
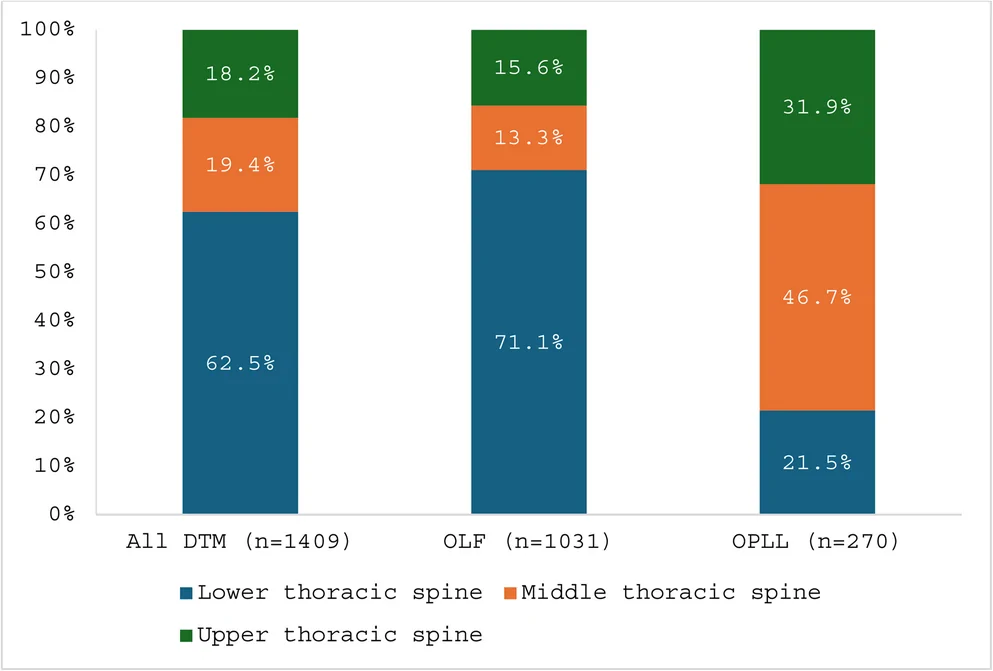
Anatomical distribution across all patients with DTM, patients with DTM secondary to OLF and patients with DTM secondary to OPLL

In total, 39 studies [2, 6, 7, 19, 25, 30, 33, 35, 37–39, 44, 45, 47, 49, 52, 53, 61, 64, 66, 68–70, 73, 76, 77, 79–81, 83, 85–87, 89, 90, 92, 93, 98] reported on the anatomical distribution of maximum spinal cord compression (Fig. 3). In 18.2% (256/1409) of patients this occurred in the upper thoracic spine (T1-T4), in 19.4% (273/1409) in the middle thoracic spine (T5-T8), and in 62.5% (880/1409) in the lower thoracic spine (T9-T12).

### Ossification of the ligamentum flavum

OLF was reported in 71.4% (2065/2893) of patients. A total of 9.2% (266/2893) of patients had concurrent OPLL and OLF. Among the 26 studies [2, 3, 6, 7, 19, 35, 39, 44, 45, 47, 49, 52, 53, 61, 64, 66, 69, 76, 81, 86–90, 93, 98] that specifically reported on the anatomical distribution of maximum spinal compression caused by OLF, the upper thoracic spine was affected in 15.6% (161/1031), the middle thoracic spine in 13.3% (137/1031) and the lower thoracic spine in 71.1% (733/1031) (Fig. 3). A total of 48.2% of OLF cases (285/591) were single level and 51.8% (306/591) were multilevel. For multilevel OLF, two vertebral levels were affected in 51.1% (95/186) of patients and more than two levels were affected in 48.9% (91/186) of patients. A total of 14 studies [6, 19, 35, 39, 41, 43–45, 69, 76, 86, 88, 96, 97] assessed OLF morphology using four different classification systems (Supplementary Materials, Table 2).

### Ossification of the posterior longitudinal ligament

OPLL was reported in 19.7% (569/2893) of patients. Eight studies [37, 38, 68, 73, 77, 79, 80, 85] specifically reported on the anatomical distribution of maximum spinal compression caused by OPLL (Fig. 3). The upper thoracic spine was affected in 31.9% (86/270), middle thoracic spine in 46.7% (126/270) and lower thoracic spine in 21.5% (58/270) of patients. In total, OPLL involved a single vertebral level in 35.1% (74/211) of patients, whilst 64.9% (137/211) of patients had multilevel involvement. Of those with multilevel OPLL, 31.1% (33/106) had two vertebral levels affected and 68.9% (73/106) had more than two vertebral levels affected. Nine studies [28, 29, 37, 38, 68, 73, 77, 79, 80] assessed OPLL morphology using two different classification systems (Supplementary Materials, Table 3).

### Intervertebral disc herniation

Intervertebral disc herniation was described in 20.1% (582/2893) of patients. Disc herniation occurred concurrently with other degenerative thoracic pathology in 14.9% (87/582) of cases: the most common concurrent pathology was OLF in 86.2% of cases (75/87), followed by calcification of the PLL in 9.2% (8/87), OPLL in 2.3% (2/87) and thoracic vertebral compression fractures in 2.3% (2/87).

### Calcification

Calcified herniated discs were reported in 2.7% (77/2893) of patients; 13.2% (77/582) of all herniated discs identified on MRI were calcified. Calcified spinal ligaments were less commonly reported and always coexisted with calcification of herniated discs. There were eight cases of PLL calcification (8/569, 1.4% of all OPLL) and a single case of calcification of the ligamentum flavum.

### Spinal cord signal change

Preoperative spinal cord signal change was identified in 30.0% (881/2940) of patients. This was described as high or increased T2 signal intensity in 90.4% (797/881). Among the remaining 84 cases, 73.8% (62/84) were described as intramedullary signal change and 26.2% (22/84) were reported as hypointense lesions.

### Spinal canal compromise

Measures of spinal canal compromise were described in 55.5% of patients (1631/2940). There was heterogeneity in the description and quantification of spinal canal compromise. Qualitative approaches involved judgement by a radiologist, including the use of the semi-quantitative Naganawa scale, where stenosis is graded from grade 0 (no stenosis) to grade 3 (severe stenosis) [56]. Three studies referred to the Naganawa grade of spinal canal compromise [22, 41, 70]. One study reported only the median grade of stenosis, which was grade three [70]. The other two studies reported on 145 patients: 0% were grade one, 2.6% (4/145) grade two, 46.9% (68/145) were grade three and 50.3% (73/145) were grade four^51,61^. The remaining studies [7, 10, 22, 28, 29, 34, 37, 38, 44, 49, 64, 68, 77, 79, 80, 82, 85, 87, 90, 94, 95, 97] quantified compromise of the spinal canal by measuring the canal diameter ratio (CDR) between the level of maximum stenosis and an unaffected part of the canal or the cross-sectional area (CSA) and anteroposterior diameter (APD) at the point of maximum stenosis. Stenosis without further quantification was described in 24 patients in two studies [72, 81].

### Single versus multilevel disease

**Fig. 4:**
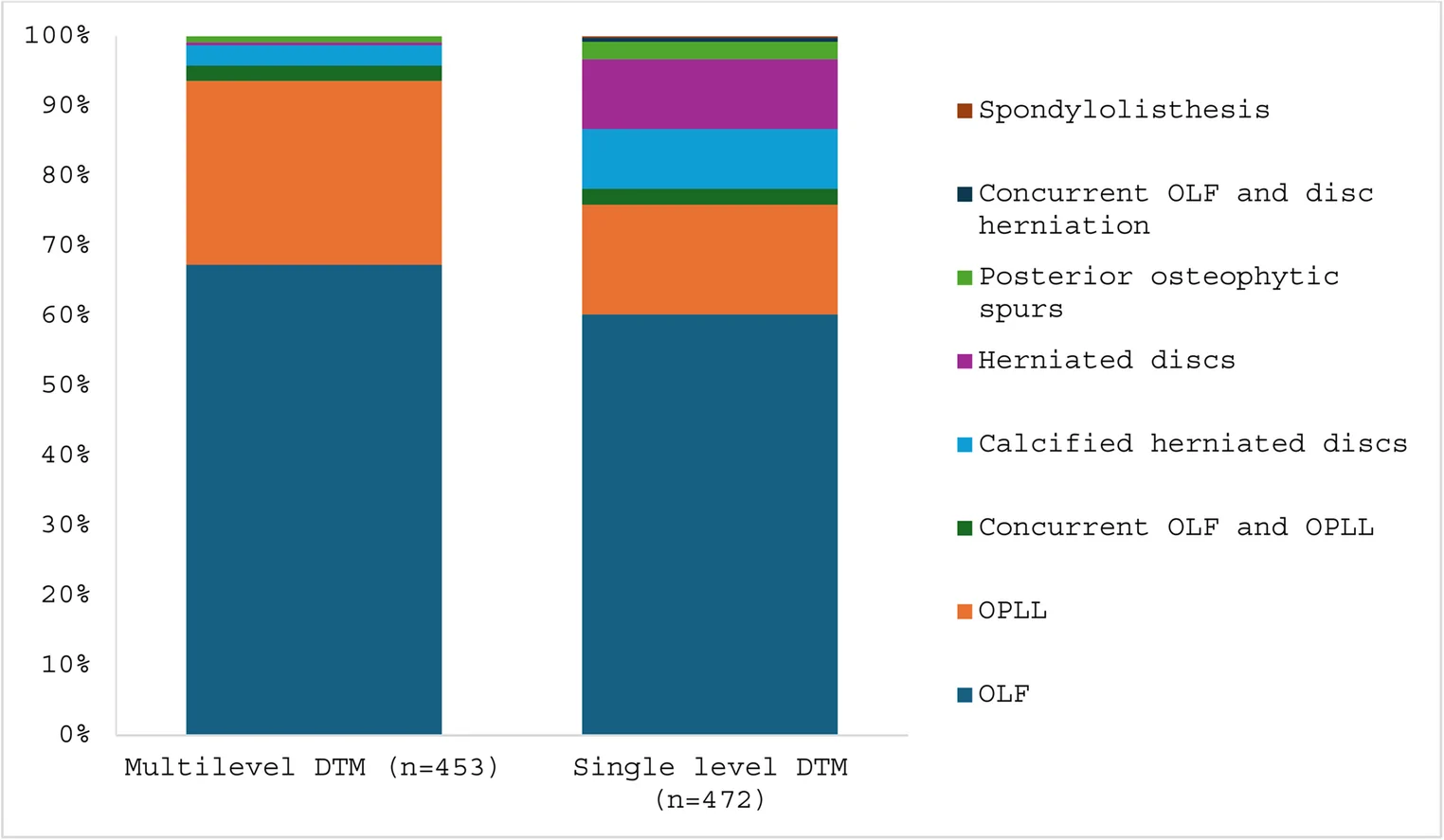
Distribution of MRI findings reported in multilevel and single level DTM

The number of vertebral levels affected was reported in 32% (925/2893) of patients: 51% (472/925) had single level and 49% (453/925) had multilevel disease (Fig. 4). In studies that specified the number of levels affected in multilevel disease, two vertebral levels were involved in 43.4% (126/290) of patients and more than two levels in 56.6% (164/290) of patients.

## Clinical significance

Associations between MRI findings and clinical outcome measures, both at baseline and postoperatively, were described in 52.5% (31/59) of studies (Supplementary Materials, Table 4).

### Spinal cord signal change

Associations with spinal cord hyperintensity were analysed in 21 studies across 1248 patients. Thirteen studies with a total of 801 included patients identified that higher grade intramedullary signal intensity correlated with a poorer recovery rate [4, 7, 14, 28, 35, 37, 39, 41, 43–45, 66, 69, 83, 93, 96–98]. Four studies with 193 included patients found that higher grade intramedullary signal intensity was not significantly correlated with recovery rate [32, 38, 53, 93]. Seven studies with a total of 415 patients identified an association between higher grade of intramedullary signal intensity and greater disease severity, measured using the modified Japanese Orthopaedic Association (mJOA) score [4, 14, 35, 37, 41, 69, 96]. One study with 77 patients found no association between signal intensity change and disease severity [7]. Four studies with 208 included patients reported an association between intramedullary signal hyperintensity and adverse surgical outcomes [29, 40, 43, 96], including spinal cord injury. Finally, one study of 118 patients reported that greater intramedullary signal hyperintensity was associated with poorer post-operative quality of life [43].

### Spinal canal compromise

Associations with spinal canal compromise were analysed in 15 studies across 1187 patients [4, 22, 29, 38, 41, 43, 44, 68, 69, 72, 80, 87, 93, 94, 97]. Four studies with 258 patients reported a positive correlation between higher grade of canal compromise and increased disease severity measured using the mJOA [22, 41, 69, 72]. One study with 46 patients found no correlation between extent of canal compromise and disease severity [4]. Six studies with a total of 372 patients found that a higher grade of canal compromise correlated with a poorer recovery rate [44, 69, 80, 87, 93, 97]. One study with 55 patients found no association between recovery rate and extent of canal compromise [68]. Correlations between spinal canal compromise and poorer surgical outcomes were identified in three studies with 152 patients [29, 38, 80]. Spinal canal compromise was also associated with poorer quality of life measured using the EQ-5D-5 L assessment in one study of 118 patients and more impaired neurological status in another study of 257 patients [43, 94].

### OLF presence and morphology

The clinical relevance of the presence of OLF and its morphology was reported across nine studies with a total of 594 patients [19, 29, 32, 40, 44, 45, 61, 93, 97]. Of these, five studies with 427 included patients reported associations between different OLF morphological types and recovery rates [19, 32, 45, 93, 97], with a fused morphology correlated with a poorer recovery rate. However, one study with 30 patients found no association between OLF morphology and recovery rate [44]. Both the presence of OLF, compared to DTM patients without OLF, and a bridged morphology, compared to OLF patients with other morphology types, correlated with poorer surgical outcome in three studies with 284 included patients [29, 62, 97]. Surgical outcome was based on a combination of patient satisfaction and change in mJOA scores postoperatively in these studies. Another study with 284 included patients reported significant differences in the postoperative mJOA score between different OLF types using a different classification system [19]. Heterogeneity in the systems used to classify OLF morphology makes it difficult to generalise these findings.

### OPLL presence and morphology

There were five studies that identified correlations between the presence and/or type of OPLL and clinical outcomes, with 268 included patients in total [4, 28, 29, 38, 80]. Of these, two studies with 106 patients found that presence of OPLL and an increased anteroposterior extent of OPLL were both associated with poorer surgical outcomes, based on the relative changes in their postoperative mJOA scores [29, 38]. There were conflicting findings regarding the clinical significance of beak-type morphology; one study with 70 patients identified it as a risk factor for poor surgical outcomes [28], whilst another with 46 patients found beak-type morphology over-represented in the group with a better recovery rate of their mJOA score [80]. Two studies, with a total of 81 patients, did not find a correlation between OPLL morphology and clinical outcomes of recovery rate and disease severity, respectively [4, 38].

### Location and extent of disease

The clinical importance of the location and/or extent of disease was investigated in 12 studies, with 766 included patients [3, 14, 32, 37, 39, 45, 53, 66, 69, 93, 97, 98]. Of these, three studies with a total of 283 patients reported a correlation between the length of spinal canal affected by degenerative disease and poorer recovery rate [14, 93, 97]. In one study of 25 patients, disease affecting single vertebral levels had better pre- and postoperative function compared to multilevel disease, assessed using the mJOA scale [37]. Another study with 57 included patients described a positive correlation between the number of affected segments and a poorer surgical outcome [66]. However, 8 studies, with 479 included patients failed to identify a correlation between the number of affected levels and the reported recovery rate [3, 32, 37, 39, 45, 53, 93, 98]. Another study with 25 patients also did not find an association between the longitudinal extent of disease and mJOA score [69].

### Risk of bias

In total, 45 studies were assessed as low risk while 14 studies were assessed as moderate risk of bias (Supplementary Materials, Figs. 1 and 2). The identification of confounders and subsequent strategies to deal with them were significant sources of bias in both the cross-sectional and cohort studies. Most included studies were observational, making them vulnerable to confounding. Furthermore, inadequately defined selection criteria and insufficient reporting of the duration of follow up were additional sources of bias in cohort studies.

## Discussion

### Summary of key findings

This review aimed to synthesise the current evidence on the spectrum of MRI findings in DTM and associations with clinical outcome measures. DTM is most common in the lower thoracic spine and ligamentous pathology appears particularly important in its aetiology. Many studies reported a measure of spinal cord compromise and an association between radiological parameters, including spinal cord signal change, and clinical outcomes, including recovery rate and patient quality of life.

### Context of findings

An exception to the overall predominance of DTM in the lower thoracic spine (T8-T12) lies in the anatomical distribution of OPLL, which is more common in the upper and middle thoracic spine. This difference in the distribution is also evident in asymptomatic patients with degenerative thoracic spinal pathology, with one study reporting T10 as the most common level for OLF and T1 for OPLL [46]. Notably, these vertebral regions are transitional areas of spinal curvature where the range of flexion, extension and rotation changes [36, 55]. Compressive load may be particularly concentrated in transitional areas, with the relative immobility of the thoracic spinal column putting additional strain on surrounding structures including ligaments [24, 20]. Repetitive microtrauma occurring in these transitional regions may promote the replacement of elastic ligamentous fibres with hypertrophic tissue rich in fibrocartilaginous cells [42]. These cells have an increased affinity for calcium deposition, promoting endochondral ossification through the replacement of hypertrophied ligament with lamellar bone [42, 15]. These hypotheses are consistent with the role that mechanical stress is postulated to play in the development of ligamentous ossification [31, 78].

DCM is most commonly reported at C5-6 or C6-7. This is hypothesised to be due to the relatively increased motion present at these levels and the resultant forces leading to a higher occurrence of cervical disc disease [91, 27]. Thus, whilst mobility in the cervical spine predisposes to disc herniations, immobility in the thoracic spine and a concentration of forces in transitional areas may predispose to ligamentous degeneration. Therefore, differences may exist between the factors driving degenerative myelopathy at different levels of the spine. This contrast highlights the importance of considering the biomechanical basis of spinal disease as a function of its anatomical distribution.

The prevalence of ligamentous ossification appears to be a notable difference between DTM and DCM. We found that a high proportion of DTM patients have OPLL and OLF, whilst much lower rates are reported in DCM populations [58]. This discordance may reflect a relatively greater importance of ligamentous pathology in DTM, but most likely reflects the Asian predominance of DTM studies included in this review.

As seen in DCM, significant geographical variation in DTM aetiology have been described [67]. In Western populations ligamentous ossification has been described at substantially lower rates than disc herniation. The opposite pattern is seen in Chinese and Japanese populations, which are well represented in this review [67]. The Asian predominance of DTM papers contrasts to reviews of research activity in DCM, where the US and Canada have predominated [54]. Whilst DTM is less common than DCM in both North America, Europe and Asia, a greater relative prevalence of DTM in Asia may, at least partially, explain the greater number of DTM studies from Asia [26]. In particular, the high representation of Chinese and Japanese studies in this review explains the high prevalence of OPLL identified. This is concordant with previously reported prevalence from these countries [67].

A similar pattern can be seen in asymptomatic individuals with incidental degenerative thoracic spinal pathology, where thoracic OLF is significantly more common than lumbar and cervical OLF [46]. The relative frequency of ligamentous ossification in the thoracic spine may also be a consequence of reduced motion in this region [50]. This may keep elastic fibres in a narrower range during movement, providing a more stable environment for ossification to occur [91]. Furthermore, existing research on the natural history of OLF describes significant enlargement in the lower thoracic spine [75]. Those with asymptomatic thoracic OLF may progress to symptomatic DTM. This would support a pathological process where ligamentous hypertrophy predates subsequent endochondral ossification.

In addition to mechanical factors, genetic factors may contribute to ligamentous pathology in the thoracic spine. In individuals with thoracic OLF, an altered genome-wide DNA methylation profile has been described [21]. OPLL has also been associated with single nucleotide polymorphisms in the osteogenic genes *IL17RC* and *COL6A1* [84]. However, environmental factors are likely to interact with genotype to drive the DTM phenotype [67]. For instance, diets involving salted products more commonly found in Chinese and Japanese meals have been implicated in the development of spinal ligament ossification [75].

### Generalisability of findings

The conceptualisation of degenerative pathology in the thoracic spine under the umbrella term DTM is increasingly recognised and is supported by the success of a similar approach in DCM [16, 58]. As proposed in DCM [17], DTM may arise as a function of abnormal mechanical loading of the spine, alongside genetic and/or environmental predisposition. As the mechanical properties of the thoracic spine differ from those of the cervical region, the location and nature of degenerative pathology that cause compression in DTM are also different (Table 2).

**Table 2:** Comparison of the prevalence of degenerative features reported on MRI from systematic reviews in both symptomatic and asymptomatic DCM, with those found in DTM in this systematic review

Prevalence	DTM	DCM [57]	Asymptomatic degenerative cervical pathology
OPLL	19.7%	10.5%	4.1% [46]
OLF	71.4%	56.8%	0.3% [8]
Multilevel disc pathology	20.1%	89.7%	64.5% [8]

[8, 46, 57]

Dynamic mechanisms appear to be of less importance owing to the relative immobility of the thoracic spine. Moreover, a greater degree of static compression may be required to bring about myelopathy in the thoracic spine compared to the cervical spine [67]. The degree of degenerative change required in the thoracic spine to achieve sufficient static compression for myelopathy to occur may therefore contribute to the relative rarity of DTM compared to DCM [67]. It may also underlie the greater prevalence of concurrent degenerative pathology in DTM, specifically ligamentous [46], as the region lacks the dynamic compression component seen in the cervical spine. Degenerative pathologies may therefore be more likely to co-exist in the thoracic spine before resulting in myelopathy [8]. 

n contrast to DCM, in which multiple studies have failed to demonstrate a consistent correlation between the degree of radiological compression and clinical severity, our synthesis of the DTM literature reveals a correlation between compression and symptom burden. Comparable findings have been reported in experimental DCM models, where myelopathic signs can be reliably induced by imposing substantial static compression, for example through expandable spacers or washers. These apparently divergent clinico-radiological relationships between cervical and thoracic disease can be reconciled within the conceptual framework proposed by the AO Spine RECODE-DCM initiative, which defines DCM as the product of mechanical stress × spinal cord vulnerability × time [17].

Several factors may modulate this interaction. The cervical spinal cord is anatomically more mobile and mechanically exposed to shear and tensile forces generated by neck movement. These forces may act as a potent trigger for the neurodegenerative cascade underlying DCM in predisposed individuals. Cervical cord vulnerability to such mechanical triggers is likely multifactorial, shaped by genetic susceptibility, ageing, and systemic comorbidities such as diabetes, which is a recognised negative prognostic factor in DCM. This may explain epidemiological observations that only approximately 10% of individuals with radiographic cervical cord compression develop symptomatic DCM, whereas in DTM symptom severity appears more directly proportional to the degree of canal compromise.

By contrast, the thoracic spine is relatively immobile and constrained within the rib cage; thus, greater static compression may be required to reach a critical threshold sufficient to provoke myelopathic symptoms. 

These comparisons support a *threshold hypothesis* for DTM: mechanical stress × time. In this framework, intrinsic cord vulnerability is less influential and mechanical compression itself is the dominant driver of neurological impairment. Future work comparing quantitative measures of cord compression, such as canal diameter ratio or cross-sectional area between the cervical and thoracic spine could test this hypothesis. This will help to determine whether lower levels of compression suffice to elicit DCM symptoms, consistent with a higher inherent vulnerability of the cervical cord.

The ageing process is also thought to impact the behaviour of neurons in response to injury [12, 59] and the biomechanical properties of the spine in response to mechanical loading [60]. Thus, chronological or biological age may play an additional role in the natural history of DTM. This is in keeping with the proposed framework of mechanical stress and time as the key processes involved in the development of DTM.

### Limitations

As with DCM, in DTM there are similar challenges relating to inconsistent terminology impeding research synthesis and understanding of this condition. 

Substantial heterogeneity between study designs and reporting of MRI findings and clinical outcomes impeded quantitative analysis. Although most included studies identified possible confounding factors, only a minority described attempts at dealing with confounders. There was also significant heterogeneity in strategies used. Furthermore, some studies used strict inclusion criteria to prevent inclusion of patients with concurrent neurological disorders and multiple degenerative pathologies in their thoracic spine. This approach may have limited the representativeness of the resulting DTM study population [67]. Improved reporting of the criteria for exclusions would make the representativeness of study populations easier to assess.

We also observed issues regarding the interpretation of MRI. For example, many studies did not define criteria for imaging features of DTM. This limited confidence in the level of inter- and intra-observer agreement for the findings reported [9, 11, 71].

## Conclusion

Ligamentous pathologies are significantly more prevalent than disc related pathologies in the DTM literature, which may reflect a predominance of Asian studies. DTM is most common in the lower thoracic spine. Signal change in the spinal cord and extent of spinal canal compromise correlate with clinical outcomes. Standardisation of the terminology used to describe imaging features and a more consistent approach to study design are required to improve future research.

## Supplementary Information

Below is the link to the electronic supplementary material.

Supplementary Material 1

## Data Availability

The data extraction form is available from the authors on request.

## Declarations

### Disclaimer

This report is independent research. The views expressed in this publication are those of the authors and not necessarily those of the NHS, CRUK, the National Institute for Health Research or the Department of Health and Social Care.
