# Supplementary material for: A systematic review of the spectrum of magnetic resonance imaging features of degenerative thoracic myelopathy
Source: Neurosurg Rev. 2026 Oct 26;49(1):623. doi: 10.1007/s10143-026-04535-z (PMC13696608; doi:10.1007/s10143-026-04535-z)
Supplement: Supplementary file 1 [file 10143_2026_4535_MOESM1_ESM.docx]

**Supplementary Materials**

**Table 1.** Search strategy used to identify papers relevant to this review in (A) Ovid MEDLINE and (B) Embase, from inception to February 13, 2025

**A)**

| **Ovid MEDLINE(R) and Epub Ahead of Print, In-Process, In-Data-Review & Other Non-Indexed Citations, Daily and Versions <1946 to February 13, 2025>** | | **Results** |
| --- | --- | --- |
| 1 | exp Thoracic Vertebrae/ or thora*.mp. | 378789 |
| 2 | exp spinal cord compression/ or exp spinal cord injuries/ or myelo*.mp. or spondylomyelo*.mp. or (spinal cord adj3 injur*).mp. or (Spinal Cord adj3 Compress*).mp. or exp Myelography/ | 465308 |
| 3 | degen*.mp. | 3118571 |
| 4 | exp Spondylosis/ or exp Osteoarthritis/ or exp Zygapophyseal Joint/ or exp Spinal Stenosis/ or exp Joint Diseases/ or exp Spondylolisthesis/ or exp Joint Instability/ or exp Intervertebral Disc Displacement/ or exp Intervertebral Disc/ or exp Intervertebral Disc Degeneration/ or exp Spinal Osteophytosis/ or arthropathy.mp. or instability.mp. or facet joint*.mp. or spondyl*.mp. or vertebral dis*.mp. or ((intervertebral dis* adj3 prolapse) or herniation or degeneration or protrusion or sequestration).mp. or osteo*.mp. or lax*.mp. or hypermobile*.mp. | 1434506 |
| 5 | exp Ehlers-Danlos Syndrome/ or exp Scheuermann Disease/ or Ehlers Danlos Syndrome.mp. or Scheuermann Disease.mp. | 5930 |
| 6 | exp Ligaments/ or exp Ossification of Posterior Longitudinal Ligament/ or exp Ligamentum Flavum/ or exp Longitudinal Ligaments/ or ((ligament adj2 calc*) or hypertroph* or ossif*).mp. or ligament* flavum.mp. or yellow ligament*.mp. or posterior longitudinal ligament*.mp. | 245151 |
| 7 | 3 or 4 or 5 or 6 | 1715367 |
| 8 | 1 and 2 and 7 | 3971 |

**B)**

| **Embase <1974 to 2025 February 13>** | | **Results** |
| --- | --- | --- |
| 1 | exp thoracic vertebra/ or exp thoracic spine/ or exp thoracic spinal cord/ or thora*.mp. | 833271 |
| 2 | exp Spinal cord compression/ or exp Spinal cord injury/ or exp Myelopathy/ or exp Myeloradiculopathy/ or myelo*.mp. or spondylomyelo*.mp. or (Spinal Cord adj3 Compress*).mp. or (spinal cord adj3 injur*).mp. | 964725 |
| 3 | exp degeneration/ or Degen*.mp. | 1299521 |
| 4 | exp Spondylosis/ or exp osteoarthritis/ or exp Spinal Osteoarthritis/ or exp Zygapophyseal Joint/ or exp Vertebral canal stenosis/ or exp Spondylosis deformans/ or exp joint degeneration/ or exp Arthropathy/ or exp joint instability/ or exp spondylolisthesis/ or exp intervertebral disk hernia/ or exp intervertebral disk/ or exp intervertebral disk degeneration/ or exp osteophyte/ or arthropathy.mp. or instability.mp. or facet joint*.mp. or spondyl*.mp. or vertebral dis*.mp. or ((intervertebral dis* adj3 prolapse) or herniation or degeneration or protrusion or sequestration).mp. or osteo*.mp. or lax*.mp. or hypermobile*.mp. | 2302261 |
| 5 | exp Ehlers-Danlos Syndrome/ or exp Scheuermann Disease/ or exp Joint hypermobility/ or exp joint laxity/ or Ehlers-Danlos Syndrome.mp. or Scheuermann Disease.mp. | 15503 |
| 6 | exp ligament/ or exp Ligament calcinosis/ or exp ligamentum flavum/ or exp Posterior longitudinal ligament/ or ((ligament adj2 calc*) or hypertroph* or ossif*).mp. or ligament* flavum.mp. or yellow ligament*.mp. or posterior longitudinal ligament*.mp. | 467054 |
| 7 | 3 or 4 or 5 or 6 | 3382352 |
| 8 | 1 and 2 and 7 | 5724 |

**Table 2.** Summary of the classification of OLF morphology according to four different classification systems.

| **Classification System** | **Morphological classification** | | | |
| --- | --- | --- | --- | --- |
| **Round or beaked morphology (n=560)** | Round | | Beaked | |
|  | 361 (64.5%) | | 199 (35.5%) | |
| **Fused or non-fused morphology (n=47)** | Fused | | Non-fused | |
|  | 24 (51.1%) | | 23 (48.9%) | |
| **Unilateral, bilateral or bridged morphology (n=63)** | Unilateral | Bilateral | | Bridged |
|  | 16 (25.4%) | 27 (42.9%) | | 20 (31.7%) |
| **Sato classification: lateral, extended or fused morphology (n=24)** | Lateral | Extended | | Fused |
|  | 4 (16.7%) | 13 (54.2%) | | 7 (29.2%) |

**Table 3.** Summary of the classification of OPLL morphology according to two different classification systems.

| **Classification System** | **Morphological classification** | | | | | | |
| --- | --- | --- | --- | --- | --- | --- | --- |
| **Flat, beaked or mixed morphology (n=128)** | Flat | | Beaked | | | Mixed | |
|  | 21 (24.2%) | | 81 (63.3%) | | | 16 (12.5%) | |
| **Sakou classification system: beaked, mixed, linear, continuous waveform, continuous cylindrical (n=236)** | Beaked | Mixed | | Linear | Continuous Waveform | | Continuous Cylindrical |
|  | 176 (74.6%) | 28 (11.9%) | | 3 (1.3%) | 18 (7.6%) | | 11 (4.7%) |

**Table 4.** Summary of findings supporting an association between MRI findings in DTM and clinical outcomes

| **MRI Pathology** | **Author (Year)** | **Sample size** | **Association with clinical outcome** | | | | |
| --- | --- | --- | --- | --- | --- | --- | --- |
|  |  |  | **Disease severity** | **Recovery rate** | **Surgical outcome** | **Neurological status** | **QoL** |
| **OLF** | Du, 2022 | 48 |  | There was a significant difference in recovery rates between OLF types in the sagittal plane. |  | There was a significant difference in postoperative JOA score between OLF types in the sagittal plane. |  |
|  | Imagama, 2017 | 70 |  |  | An absence of OLF, spinal cord hyperintensity and OPLL at the same level was a predictor of a good surgical outcome. |  |  |
|  | Kang, 2011 | 51 |  | Significantly better recovery rate with beak type OLF compared to round. | Beak type OLF was a predictor of a better surgical outcome and patient satisfaction grade postoperatively. |  |  |
|  | Li, 2016 | 85 |  | Recovery rate correlated significantly with axial configurations of OLF. |  |  |  |
|  | Pan, 2023 | 48 |  |  | Dural ossification (DO) was predominantly found in fused and tuberous cases of OLF, with a previously noted association between DO and surgical complications. |  |  |
|  | Sung, 2006 | 19 |  |  | Beak type OLF lesion was a predictor of a worse postoperative surgical outcome. |  |  |
|  | Yu, 2013 | 78 |  | Fused-type OLF was related to a worse recovery rate. |  |  |  |
|  | Zhang, 2021 | 165 |  | The ‘poor recovery’ group had significant differences in the type of OLF compared to the ‘good outcome’ group. | Bridged type OLF was a significant independent risk factor for predicting poor preliminary postoperative outcome. |  |  |
| **OPLL** | Imagama, 2017 | 70 |  |  | Beak-type OPLL was identified as a preoperative risk factor for poor surgical outcome. |  |  |
|  | Imagama, 2017 | 71 |  |  | Absence of OPLL, OLF and spinal cord hyperintensity at the same level was a predictor of good surgical outcome. |  |  |
|  | Ko, 2020 | 35 |  |  | The anteroposterior length of the OPLL at the maximally affected level was associated with surgical outcome. |  |  |
|  | Ushio, 2023 | 46 |  | The proportion of patients with beak-type OPLL was significantly higher in the >50% JOA improvement group. |  |  |  |
| **Spinal cord hyperintensity** | Ando, 2017 | 46 | The group diagnosed with ‘an absence of or mild myelopathy’ despite OPLL had significantly lower rates of spinal cord hyperintensity than the group with a JOA score of 7 or more. | Statistically significant relationship between recovery rate and spinal cord signal intensity. |  |  |  |
|  | Bagga, 2023 | 77 |  | Absence of spinal cord signal intensity on MRI significantly associated with better recovery rate. |  |  |  |
|  | Cao, 2018 | 40 | Compared to the group with high-intensity spinal cord changes, the group with low-intensity spinal cord changes had a significantly lower proportion with severe thoracic spinal stenosis and a higher preoperative JOA score. | Post-operative recovery rate was lower in group with hyper-intense spinal cord changes. |  |  |  |
|  | Imagama, 2017 | 71 |  |  | Predictors of good surgical outcome was lower rates of hyperintensity areas, whilst factors related to poor surgical outcome included spinal cord damage. |  |  |
|  | Kim, 2018 | 24 | Significant correlation between preoperative JOA and signal intensity ratio. | Significant correlations between signal intensity ratio and JOA RR. SIT was a significant contributor for predicting JOA RR. |  |  |  |
|  | Kim, 2020 | 25 | High intramedullary signal intensity on preoperative MRI associated with significantly lower preoperative JOA scores. | Patients with intramedullary high signal intensity on preoperative MRI shower significantly lower recovery rates than patients without. |  |  |  |
|  | Lee, 2019 | 161 | Significantly more patients with hyperintensity changes on MRI in symptomatic OLF group compared to asymptomatic OLF group. |  |  |  |  |
|  | Li, 2016 | 85 |  | Intramedullary signal change correlated significantly with the recovery rate. |  |  |  |
|  | Li, 2020 | 30 |  | A poor recovery rate was significantly related to the presence of high intensity signal. |  |  |  |
|  | Li, 2004 | 24 |  |  | Intramedullary signal size showed a significant correlation with postoperative overall QoL postoperatively. In patients with moderate/mild neurological injury, there was a significantly lower QoL in patients with increased signal cord signal which also showed significant correlation with the mobility dimension of QoL. |  |  |
|  | Ranjan, 2024 | 57 |  |  |  | Intramedullary signal intensity was significantly associated with worse surgical outcome. |  |
|  | Sanghvi, 2011 | 25 | Preoperative intramedullary signal size significantly correlated with preoperative neurological status. | Preoperative intramedullary signal size correlated significantly with recovery rate. |  |  |  |
|  | Kuh, 2006 | 19 |  |  | Preoperative intramedullary signal change was a predictor of worse postoperative surgical outcome. |  |  |
|  | Wang, 2013 | 16 |  | Significant differences in recovery rate between the group where ISI disappeared and the group where it was continuous. Group with continuously positive ISI showed a poor recovery rate compared to the ISI-negative group and the postoperative ISI-negative group that had essentially the same recovery rates. |  |  |  |
|  | Yu, 2013 | 78 |  | Recovery rate correlated significantly with the intramedullary signal intensity. |  |  |  |
|  | Zhang, 2016 | 96 |  | Preoperative signal change ratio correlated with JOA recovery rate; was significantly greater in poor recovery rate than in the good recovery rate. |  |  |  |
|  | Zhang, 2021 | 165 |  | Poor recovery group had a longer extension of increased signal on sagittal T2I as well as an association with type of increased signal on axial T2 images. The grade of ISST2I was a significant independent risk factor for predicting poor preliminary postoperative outcome |  |  |  |
|  | Zhang, 2023 | 94 | Higher grade of intramedullary signal intensity was significantly associated with more frequent neurological symptoms, longer duration of symptoms and worse preoperative JOA score. | Higher grade of intramedullary signal intensity was associated with a worse postoperative recovery at follow-up. | Higher grade of intramedullary signal intensity associated with significantly increased operation time, intraoperative blood loss, CSF leakage and wound infection. |  |  |
| **Spinal canal compromise** | Feng, 2015 | 44 | Grade of canal occupation significantly correlated with preoperative JOA score.  Asymptomatic controls had significantly lower grades of spinal canal occupation. |  |  |  |  |
|  | Imagama, 2017 | 71 |  |  | The ‘good outcome’ group had lower measures of spinal cord stenosis. |  |  |
|  | Ko, 2020 | 35 |  |  | The rate of spinal occupancy at the maximally affected level was significantly associated with surgical outcome. |  |  |
|  | Lee, 2019 | 161 | Significantly greater grade of canal compromise in the symptomatic group compared to the asymptomatic group. |  |  |  |  |
|  | Li, 2020 | 30 |  | A poor recovery rate was significantly related to a lower ratio of APD and CSA. |  |  |  |
|  | Li, 2004 | 24 |  |  |  |  | • A spinal occupation rate of >50% showed a significant correlation with overall QoL postoperatively. |
|  | Sanghvi, 2011 | 25 | Preoperative canal grade significantly correlated with preoperative neurological status. | Preoperative canal grade is significantly correlated with recovery rate. |  |  |  |
|  | Sugimura, 1992 | 28 | There was a strong relationship between the grade of MRI cord compression and degree of myelopathy |  |  |  |  |
|  | Ushio, 2023 | 46 |  | The canal occupancy ration was significantly lower in the >50% JOA improvement group than in the less than or equal to 50% group. | Low canal occupancy ratio was a predictor of good surgical outcome. |  |  |
|  | Wu, 2021 | 28 |  | There is a correlation between statistically significant improvement in JOA score and the extent of MRI CSA increase postoperatively. |  |  |  |
|  | Yu, 2013 | 78 |  | Recovery rate significantly correlated with the degree of compression in the APD. |  |  |  |
|  | Yuan, 2021 | 257 |  |  |  | Higher preoperative spinal canal occupancy was identified as a risk factor for postoperative neurological deterioration. |  |
|  | Zhang, 2021 | 165 |  | Poor recovery group had significantly higher spinal canal occupation ratio. |  |  |  |
| **Extent of disease (single or multi-level)** | Cao, 2018 | 40 |  | The rate of postoperative recovery was significantly lower for patients with increased spinal cord intensity for multiple segments compared to those with increased signal intensity for a single segment. |  |  |  |
|  | Kim, 2020 | 25 |  |  |  |  | • Single level lesions had higher pre and postoperatively JOA scores. |
|  | Ranjan, 2024 | 57 |  |  | Multiple segment involvement of the OLF was significantly associated with a worse surgical outcome. |  |  |
|  | Sung, 2006 | 19 |  |  | A single level OLF lesion was correlated with a ‘good’ postoperative surgical outcome. |  |  |
|  | Yu, 2013 | 78 |  | The mean recovery rate correlated significantly with the number of segments and regions involved. |  |  |  |
|  | Zhang, 2021 | 165 |  | The ‘poor recovery’ group had significantly longer extension of spinal cord hyperintensity than the ‘good recovery’ group. |  |  |  |
| **Location of disease** | Li, 2016 | 85 |  | Recovery rate correlated significantly with the OLF level, whether upper, middle or lower thoracic spine. |  |  |  |
|  | Yu, 2013 | 78 |  | The mean recovery rate was significantly worse in OLF found in the middle thoracic spine. |  |  |  |

**Figure 1.** Risk of bias assessment**.** Histogram of distribution of per-item scores for cross-sectional studies, indicating for each item, the number of articles scoring ‘Yes’, ‘Unclear’, ‘No’, or ‘N/A’. NB: Due to a relatively high prevalence of N/A answers to different questions, resulting from heterogeneity in the design of included cohort studies, the aggregate scores are displayed as percentages.

Cross-Sectional Studies Per-Item Score

Number of studies

|  | Yes | Unclear | No | N/A |
| --- | --- | --- | --- | --- |
| Selection Criteria | 2 | 1 | 2 | 0 |
| Subject and Setting Description | 5 | 0 | 0 | 0 |
| Valid Exposure Measurement | 5 | 0 | 0 | 0 |
| Objective Condition Measurement | 4 | 1 | 0 | 0 |
| Identifying Confounders | 3 | 0 | 2 | 0 |
| Dealing with Confounders | 0 | 0 | 5 | 0 |
| Valid Outcome Measurements | 2 | 2 | 1 | 0 |
| Statistical Analysis | 3 | 0 | 2 | 0 |

**Figure 2.** Risk of bias assessment. Histogram of distribution of per-item scores for cohort studies, indicating for each item, the number of articles scoring ‘Yes’, ‘Unclear’, ‘No’, or ‘N/A’. NB: Due to a relatively high prevalence of N/A answers to different questions, resulting from heterogeneity in the design of included cohort studies, the aggregate scores are displayed as percentages.

Cohort Studies Per-Item Score

Number of studies

|  | Yes | Unclear | No | N/A |
| --- | --- | --- | --- | --- |
| Cohort Similarity | 23 | 0 | 0 | 35 |
| Similar Exposure Measurements | 0 | 0 | 0 | 58 |
| Valid exposure Measurements | 0 | 0 | 0 | 58 |
| Identifying Confounders | 40 | 9 | 9 | 0 |
| Dealing with Confounders | 24 | 3 | 31 | 0 |
| Exposure Free at Start | 52 | 0 | 0 | 6 |
| Valid Outcome Measurements | 52 | 4 | 0 | 2 |
| Follow Up Length | 38 | 3 | 13 | 4 |
| Loss to Follow Up | 31 | 6 | 2 | 19 |
| Dealing with Loss to Follow Uo | 1 | 3 | 7 | 47 |
| Statistical Analysis | 50 | 0 | 8 | 0 |
